# Deep learning-based prediction of cervical canal stenosis from mid-sagittal T2-weighted MRI

**DOI:** 10.1007/s00256-025-04917-2

**Published:** 2025-03-28

**Authors:** Wounsuk Rhee, Sung Cheol Park, Hyoungmin Kim, Bong-Soon Chang, Sam Yeol Chang

**Affiliations:** 1 Ministry of Health and Welfare, Government of the Republic of Korea, 13, Doum 4-Ro, Sejong, 30113 Republic of Korea; 2 Siebel School of Computing and Data Science, University of Illinois Urbana-Champaign, 201 N. Goodwin Avenue, Champaign, IL 61801 USA; 3 Healthcare AI Research Institute, Seoul National University Hospital, 101, Daehak-Ro, Jongno-Gu, Seoul, 03080 Republic of Korea; 4 Department of Orthopedic Surgery, Bumin Hospital Seoul, 389, Gonghang-daero, Gangseo-gu, Seoul, 07590 Republic of Korea; 5 Department of Orthopedic Surgery, Seoul National University College of Medicine, 103, Daehak-Ro, Jongno-Gu, Seoul, 03080 Republic of Korea; 6 Department of Orthopedic Surgery, Seoul National University Hospital, 101, Daehak-Ro, Jongno-Gu, 03080 Seoul, Republic of Korea

**Keywords:** Degenerative cervical myelopathy, Cervical canal stenosis, Deep learning, Computer vision

## Abstract

**Objective:**

This study aims to establish a large degenerative cervical myelopathy cohort and develop deep learning models for predicting cervical canal stenosis from sagittal T2-weighted MRI.

**Materials and methods:**

Data was collected retrospectively from patients who underwent a cervical spine MRI from January 2007 to December 2022 at a single institution. Ground truth labels for cervical canal stenosis were obtained from sagittal T2-weighted MRI using Kang’s grade, a four-level scoring system that classifies stenosis with the degree of subarachnoid space obliteration and cord indentation. ResNet50, VGG16, MobileNetV3, and EfficientNetV2 were trained using threefold cross-validation, and the models exhibiting the largest area under the receiver operating characteristic curve (AUC) were selected to produce the ensemble model. Gradient-weighted class activation mapping was adopted for qualitative assessment. Models that incorporate demographic features were trained, and their corresponding AUCs on the test set were evaluated.

**Results:**

Of 8676 patients, 7645 were eligible for developing deep learning models, where 6880 (mean age, 56.0 ± 14.3 years, 3480 men) were used for training while 765 (mean age, 56.5 ± 14.4 years, 386 men) were set aside for testing. The ensemble model exhibited the largest AUC of 0.95 (0.94–0.97). Accuracy was 0.875 (0.851–0.898), sensitivity was 0.885 (0.855–0.915), and specificity was 0.861 (0.824–0.898). Qualitative analyses demonstrated that the models accurately pinpoint radiologic findings suggestive of cervical canal stenosis and myelopathy. Incorporation of demographic features did not result in a gain of AUC.

**Conclusion:**

We have developed deep learning models from a large degenerative cervical myelopathy cohort and thoroughly explored their robustness and explainability.

## Introduction

Degenerative cervical myelopathy (DCM) is a general term for age-related disorders of the cervical spine that includes cervical spondylotic myelopathy and ossification of the posterior longitudinal ligament (OPLL) [1–3]. The clinical significance of DCM lies in its wide prevalence and potential to cause neurological impairments, including motor weakness, sensory deficits, and gait disturbances, that can impact daily functioning and quality of life [1, 2]. The condition often necessitates surgical intervention and extensive rehabilitation to alleviate symptoms and prevent further decline of neurological functions [1–4].

DCM is usually diagnosed by identifying cervical canal stenosis (CCS) in cervical spine MRI. Kang et al. proposed a four-level grading system that evaluates CCS from sagittal T2-weighted images based on the degree of subarachnoid space obliteration and cord indentation, reporting good to excellent inter-observer agreement (intra-class correlation [ICC], 0.74; range 0.72–0.80) and intra-observer agreement of 0.77 (0.68–0.81) [5, 6]. The agreement for binary classification of CCS (grade 0–1 vs. grade 2–3) ranged from 0.81 to 0.85, with Cohen’s kappa ranging from 0.57 to 0.66, and it has been demonstrated that such grouping methods correlate with the presence of neurological symptoms with kappa of 0.81 (0.70–0.92) [7].

Recent advancements in computer vision tasks, represented by deep learning (DL), have significantly enhanced the radiological diagnostic process by reducing diagnostic time and improving consistency, and there has also been growing attention to applying DL to biomedical images acquired from the cervical spine, including X-rays, MRI, and endoscopy [8–15]. However, this field of research is still in its infancy, and several key challenges remain. First, the cohorts used for training models are relatively small, ranging from dozens to a few hundred individuals, inherently limiting generalizability. To address this issue, preliminary studies have inevitably favored extracting multiple images from a single patient [12, 13]. In addition, most are pilot investigations applying a single architecture despite the abundance of novel models, thus failing to fully showcase the full potential of DL [10–12].

We aimed to establish a cohort five to ten times larger than those explored in preliminary studies and explore DL models that robustly predict CCS solely from sagittal T2-weighted MRI images, a topic that has not previously been well-established in the literature. Various architectures were experimented with to provide a more systematic comparison of their properties. Additionally, gradient-weighted class activation mapping (Grad-CAM) techniques were employed to assess the explainability of each model [16].

## Materials and methods

### Patient selection

Upon approval by the institutional review board (IRB) and having granted a waiver of consent (IRB number: H-2402–077–1512), anonymized data was retrospectively collected from consecutive patients who had undergone an MRI of the cervical spine from January 2007 to December 2022 at a single institution, a tertiary care hospital. Of the identified patients, individuals with a history of cervical spine operation before MRI and those diagnosed with non-degenerative conditions affecting bone and spinal cord, including infection, trauma, tumor, and deformity, were excluded. To standardize the spatial resolution of MRI, patients without T2-weighted sagittal MRI scans of 3-mm section thickness were excluded.

### Data acquisition

MRI instrument names, manufacturers, study protocols, and pixel data were extracted from the original DICOM files using Pydicom (version 2.4.4). Although patient data was collected at a single institution, study specifications varied because the dataset contained studies performed outside our organization but registered to the database for clinical purposes. The means and standard deviations of echo time (TE) and repetition time (TR) were 112 ± 15 ms and 3360 ± 710 ms, respectively, and the matrix size of the raw MRI images was mostly 512 × 512. From each patient, three non-contrast T2 weighted sagittal sections of the cervical spine around the mid-sagittal plane were manually collected: the mid-sagittal section and a pair of adjacent slices. Each grayscale image was designated to red, green, and blue channels and merged channel-wise so that the input could be accepted by pretrained image classification models. The merged images were then manually cropped and resized to a shape of 224 × 112 × 3 so that the region of interest best captures the vertebral bodies and spinous processes of the seven cervical vertebrae. To promote generalizability, we did not apply any other pixel-wise modifications, such as brightness and contrast adjustment. The whole image preprocessing procedure was done with OpenCV-Python library (version 4.9.0).

Ground truth labels for CCS were obtained from T2-weighted MRI images by a spine specialist with more than 5 years of experience in spine surgery, using Kang’s grade [6]. In this study, each case was labeled in a binary fashion, where patients with Kang grade 2–3 were designated to the CCS-positive class, and those with Kang grade 0–1 were assigned to the CCS-negative class because Kang grade 2 or higher indicates the actual compression of the spinal cord, requiring surgical intervention [5–7].

Statistical analysis comparing the distribution between the train and test sets was conducted. Continuous variables were analyzed using an independent t-test, while categorical variables were compared using the *χ*^2^ test. In our study, *p*-values under 0.05 indicate statistical significance, and analyses were carried out using SciPy (version 1.13.0) and Statsmodels (version 0.14.2).

### Model training and evaluation

The cohort was randomly split into train and test sets containing 90% and 10% of the cases, respectively, and the train set was further divided into three subgroups to perform threefold cross-validation. In each fold, images from two subgroups were expanded three times through data augmentation consisting of random rotation from − 10 to + 10° and random scaling from − 10 to + 10%. These procedures are widely accepted in practice because they promote generalizability by mitigating potential biases arising from the limited dataset. The remaining subgroup was used for internal validation, which was carried out for each epoch, and the test set was used only once per model architecture to measure the model’s performance at the final stage.

We applied transfer learning to some ImageNet-pretrained CNN models, including ResNet50, VGG16, MobileNetV3, and EfficientNetV2 [17–20]. The final layers were modified to be suited for binary classification, and pretrained layers were fine-tuned by sequentially de-freezing the model from back to front. The set of hyperparameters that resulted in the greatest area under the receiver operating characteristic curve (AUC) on the test set were identified through random search within a search space commonly accepted in practice. Finally, we propose an ensemble model in which the output is determined as the average of the output probabilities from the best-performing models from each of the four distinct architectures.

Classifier performance was evaluated on the test set using various metrics. Specifically, the AUC was used as our primary outcome measure, and auxiliary indicators, such as accuracy, sensitivity, specificity, positive predictive value (PPV), and negative predictive value (NPV), were obtained from confusion matrices generated using a decision threshold of 0.5. Moreover, we adopted Grad-CAM techniques to qualitatively assess explainability [16]. Finally, we assessed the impact of demographic features by training a logistic regression model using age, sex, and the output probabilities from each of the four deep learning models: ResNet50, VGG16, MobileNetV3, and EfficientNetV2. We then compared their AUCs on the test set with that of the ensemble model.

The whole process was carried out using TensorFlow (version 2.13.0) and Scikit-learn (version 1.4.2) on a computer with an Intel(R) Core(TM) i9-14900KF CPU, an NVIDIA RTX 4090 24GB graphics card, and 64GB of DDR5 RAM.

## Results

### Patient characteristics

**Fig. 1:**
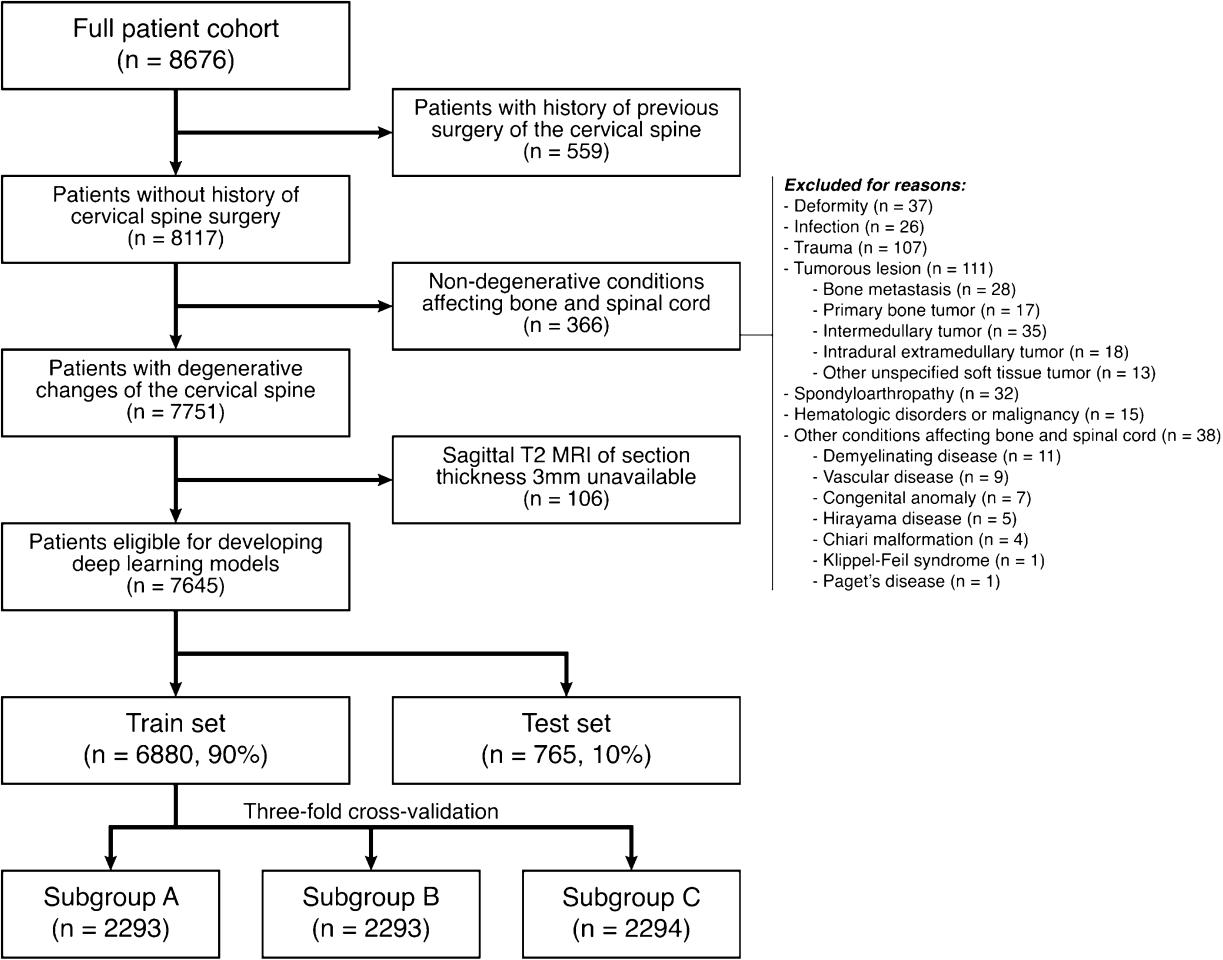
A flowchart of the patient selection and division process

Among a total of 8676 patients, 559 had a history of previous cervical spine surgery, and 366 were identified with non-degenerative conditions affecting bone and spinal cord, including tumor (*n* = 111), trauma (*n* = 107), deformity (*n* = 37), spondyloarthropathy (*n* = 32), infection (*n* = 26), hematologic disorder (*n* = 15), and other disease comprising the minority (*n* = 38). Patients without sagittal T2 MRI of section thickness 3 mm (*n* = 106) were further excluded. Consequently, 7645 individuals, each having one MRI scan, were eligible for developing DL models, and it was randomly split into train and test sets containing 6880 and 765 cases, respectively. The patient selection process is shown in Fig. 1.

Table 1 summarizes the results of statistical analysis on the demographic features of the patient cohort. We found no evidence of a difference between the train and test sets in age (56.0 ± 14.3 years vs. 56.5 ± 14.4 years; *p* = 0.42), sex (50.6% male vs. 50.5% male; *p* = 0.98), and the distribution of ground truth labels (59.3% positive vs. 56.7% positive; *p* = 0.18). The CCS-positive group was older (60.0 ± 12.7 years) than the CCS-negative group (50.6 ± 15.7 years) (*p* < 0.001) and contained more male patients (57.4% male vs. 40.7% male; *p* < 0.001).

**Table 1:** Patient characteristics of train and test sets

Features	Train set (*n* = 6880)	Test set (*n* = 765)	*p*-value*	CCS-negative (*n* = 3131)	CCS-positive (*n* = 4514)	*p*-value**
Age (years)	56.0 ± 14.3	56.5 ± 14.4	0.42	50.6 ± 15.7	60.0 ± 12.7	< 0.001
Sex			0.98			< 0.001
Male	3480	386		1274	2592	
Female	3400	379		1857	1922	
CCS			0.18			
Negative (grade 0–1)	2800	331				
Positive (grade 2–3)	4080	434				

Note: Age is presented as mean age (years) ± standard deviation, and other variables are presented as value counts. Independent *t*-tests were performed for the comparison of age distribution, and chi-square tests were performed to compare the distribution of sex and CCS labels. *CCS*, cervical canal stenosis

^*^p-value for comparing the demographic characteristics of the train and test sets

^**^p-value for comparing the demographic characteristics of CCS-negative and CCS-positive groups

### Model performance

**Fig. 2:**
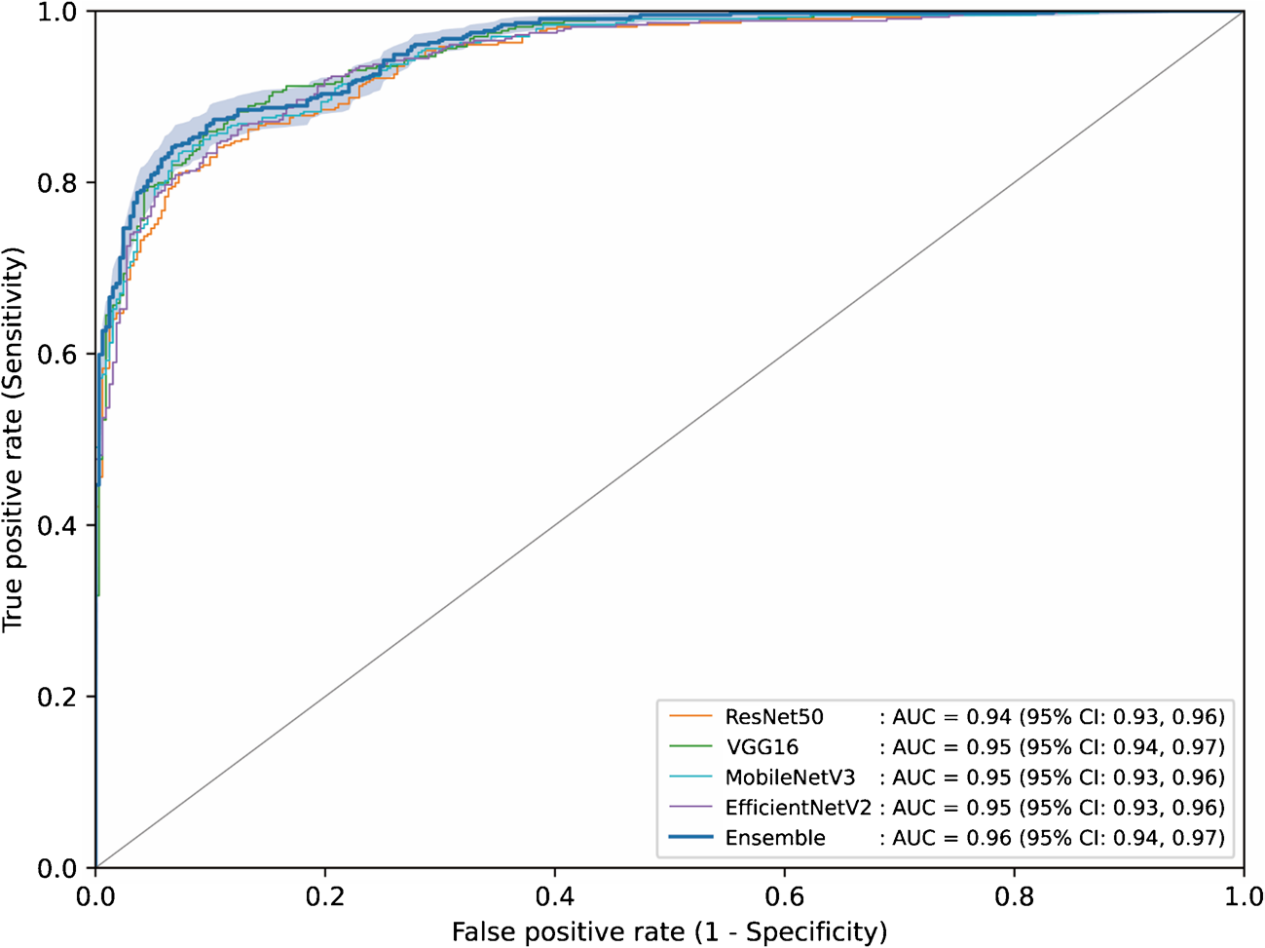
ROC curves of each model on the test set. Shaded area indicates the 95% confidence interval of the ROC curve of the ensemble model

**Fig. 3:**
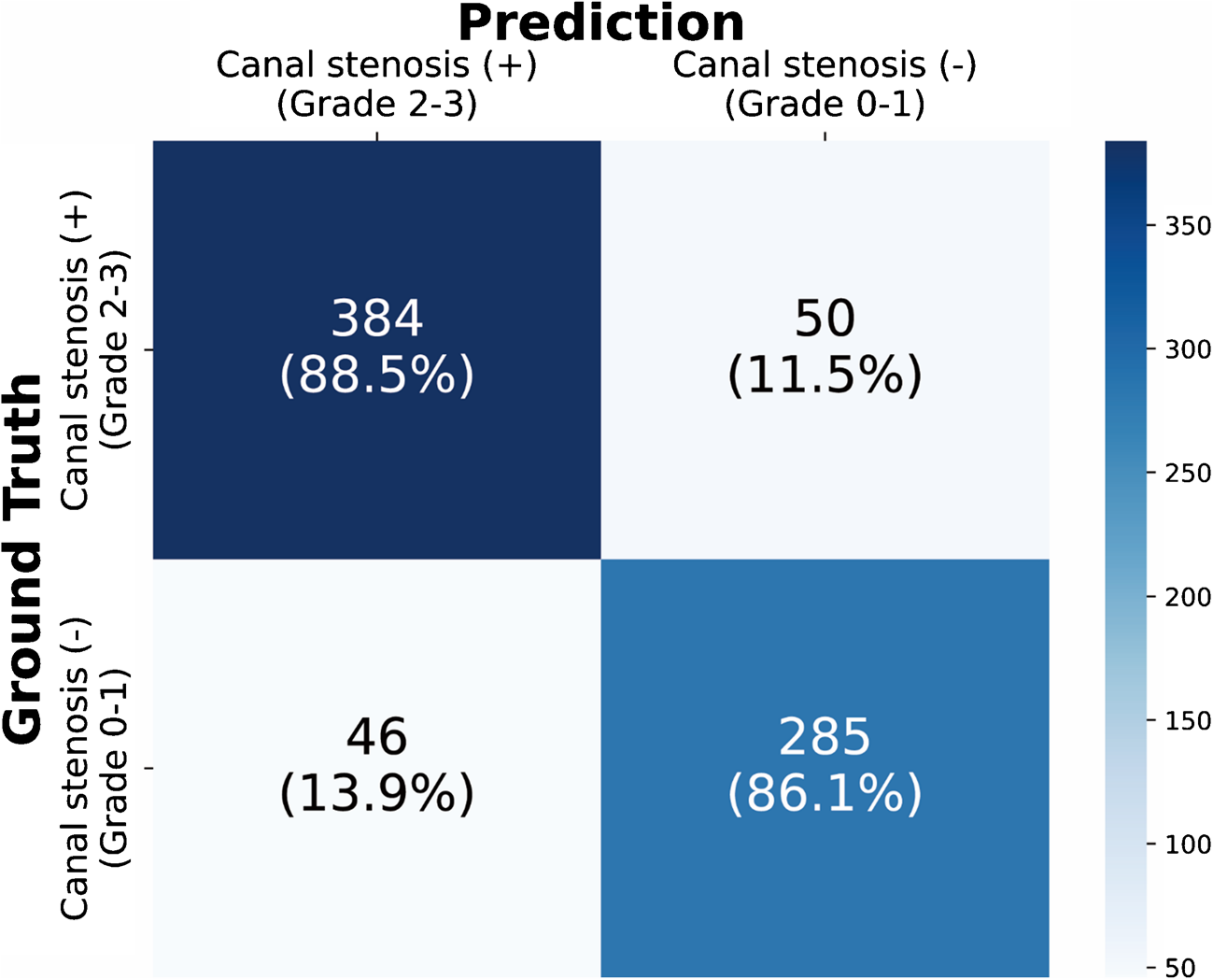
The confusion matrix of the ensemble model on the test set. It should be noted that the prediction is based on the cutoff value of 0.5

Receiver operating characteristic curves and performance metrics for ResNet50, VGG16, MobileNetV3, EfficientNetV2, and the ensemble model are shown in Fig. 2 and Table 2. The ensemble model resulted in the largest AUC(0.96; 95% CI 0.94–0.97), with an accuracy of 0.875 (95% CI 0.851–0.898), sensitivity of 0.885 (95% CI 0.855–0.915), specificity of 0.861 (95% CI 0.824–0.898), PPV of 0.893 (95% CI 0.864–0.922), and NPV of 0.851 (95% CI 0.813–0.889), in which the values were derived from the confusion matrix shown in Fig. 3.

**Table 2:** Performance of deep learning models of different architectures

Model	AUC	Accuracy	Sensitivity	Specificity	PPV	NPV
ResNet50	0.94 (0.93–0.96)	0.846 (0.820–0.871)	0.887 (0.857–0.917)	0.792 (0.748–0.835)	0.848 (0.815–0.881)	0.842 (0.802–0.883)
VGG16	0.95 (0.94–0.97)	**0.878 (0.855–0.902)**	**0.906 (0.878–0.933)**	0.843 (0.804–0.882)	0.883 (0.853–0.913)	**0.872 (0.835–0.908)**
MobileNetV3	0.95 (0.93–0.96)	0.867 (0.843–0.891)	0.869 (0.837–0.900)	0.864 (0.827–0.901)	0.893 (0.864–0.923)	0.834 (0.794–0.873)
EfficientNetV2	0.95 (0.93–0.96)	0.868 (0.844–0.892)	0.866 (0.834–0.898)	**0.870 (0.834–0.906)**	**0.897 (0.868–0.926)**	0.832 (0.793–0.872)
Ensemble model	**0.96 (0.94–0.97)**	0.875 (0.851–0.898)	0.885 (0.855–0.915)	0.861 (0.824–0.898)	0.893 (0.864–0.922)	0.851 (0.813–0.889)

Note: 95% confidence intervals are in parentheses. The greatest values for each evaluation metric are boldfaced. *AUC*, area under the receiver operating curve; *PPV*, positive predictive value; *NPV*, negative predictive value

### Grad-CAM analysis

**Fig. 4:**
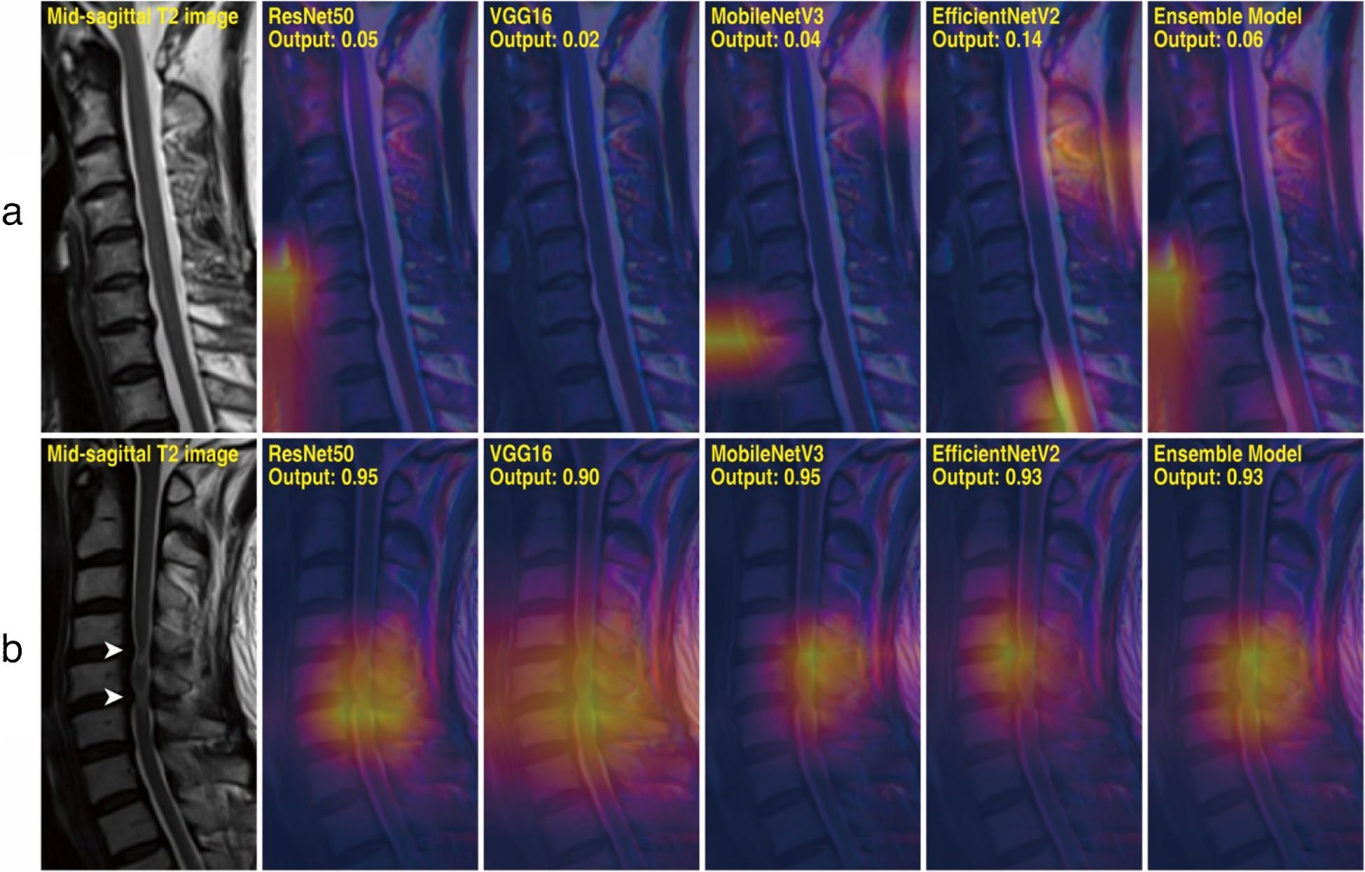
Grad-CAM results of representative cases for the negative and positive classes. Non-contrast mid-sagittal T2-weighted MRI image is shown, followed by a series of Grad-CAMs obtained from different models. **a** A 72-year-old female of Kang grade 0 with no identified cord compression. **b** A 47-year-old male of Kang grade 3 with cord compression and signal change in the spinal cord at C4-5 and C5-6 (arrowheads)

**Fig. 5:**
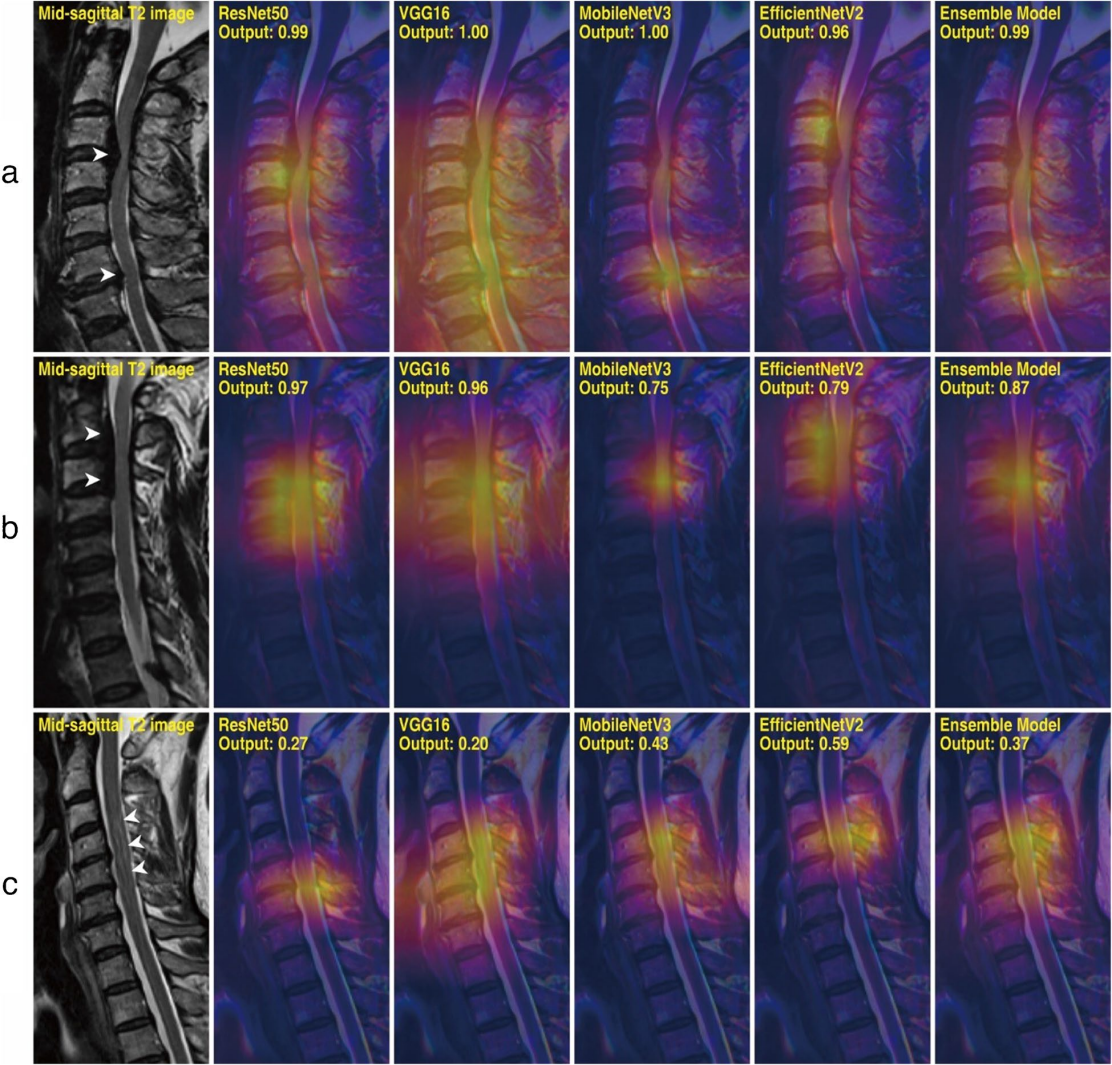
Grad-CAM results of test samples that best demonstrate the robustness of deep learning models. Non-contrast mid-sagittal T2-weighted MRI image is shown, followed by a series of Grad-CAMs obtained from different models. **a** A 66-year-old male of Kang grade 3 with multiple cervical canal stenosis at C3-4 and C6-7 (arrowheads). **b** A 64-year-old male of Kang grade 2 with cervical canal stenosis at C2-4 due to ossification of the posterior longitudinal ligament (OPLL) (arrowheads). **c** A 63-year-old female of Kang grade 1 with T2 hyperintensity in C3-6 without definite evidence of cord compression (arrowheads)

**Fig. 6:**
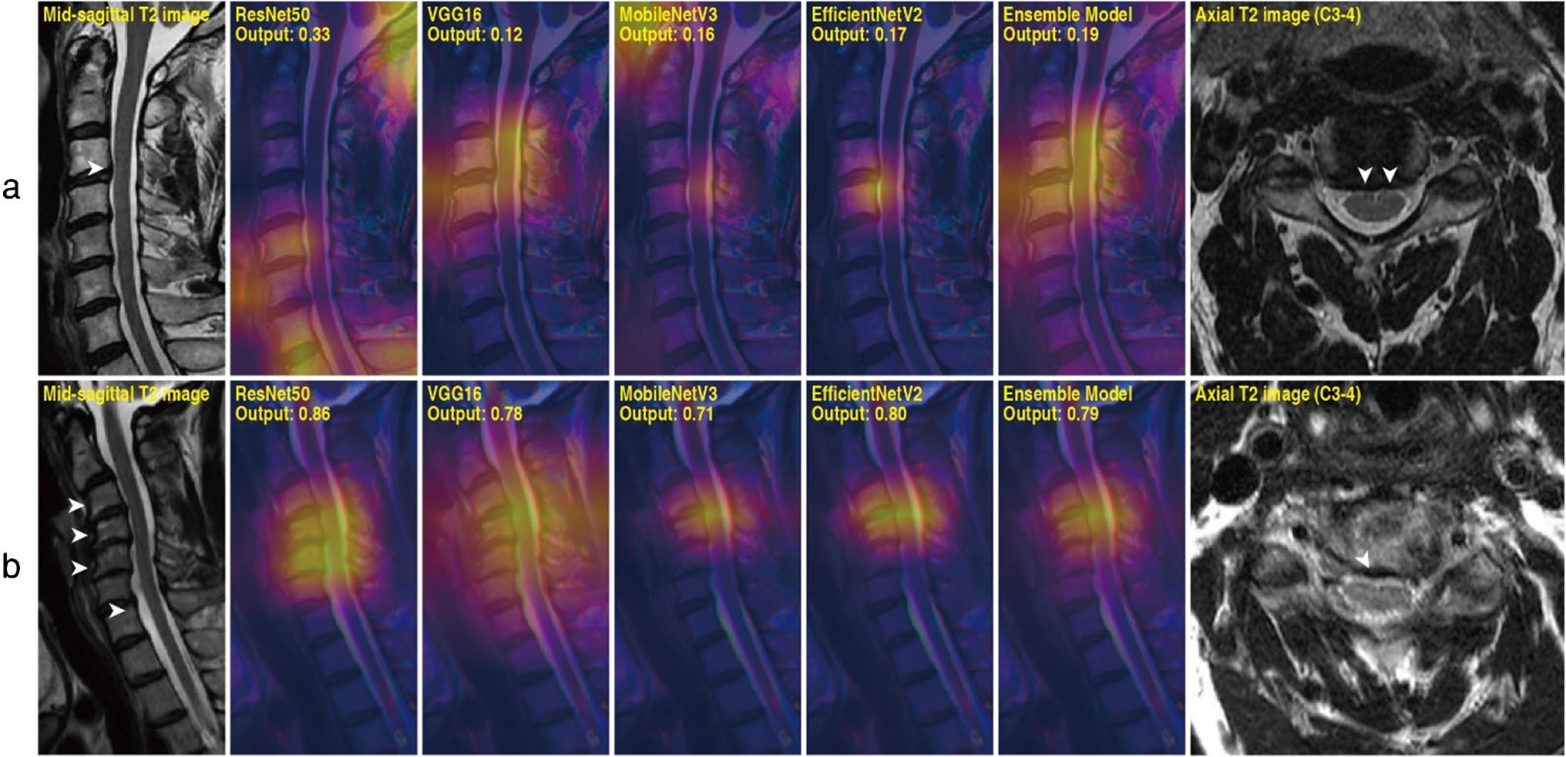
Grad-CAM results of test samples producing false predictions. Non-contrast mid-sagittal T2-weighted MRI image is shown, followed by a series of Grad-CAMs obtained from different models and an axial section of the most affected region. **a** A 62-year-old male of Kang grade 2 with spinal cord compression at C3-4 (arrowheads). **b** A 48-year-old female of Kang grade 1 with severely progressed cervical spondylosis at C3-5 (arrowheads)

Grad-CAM analysis was conducted on samples from the test set to assess the models’ explainability. Figure 4 lists a few representative cases of CCS-negative and CCS-positive patients in which the DL models produced correct results. Figure 5 provides test samples in which the robustness of the models was best highlighted, and Fig. 6 illustrates a few instances in which the ensemble model has generated false predictions. For each model in each case, a heatmap indicating relative magnitudes of gradient activations is overlapped with the input image. It should be noted that models are more inclined to focus on regions with warmer colors than other regions.

Overall, DL models effectively identified radiologic findings suggestive of CCS and myelopathy, including disc bulging, obliteration of cerebrospinal fluid (CSF) space, and increased T2 signal intensity of the spinal cord. Even in challenging cases where the models produced false predictions, as listed in Fig. 6, they attended to regions with the highest chance of finding abnormalities. Further interpretation and implications of Grad-CAM results are elaborated in the “Discussion” section.

### Effects of demographics on performance

Results of ablation studies aiming to assess the effects of demographic features on model performance are summarized in Table 3. Despite the statistically significant difference in the distribution of age and sex between CCS-positive and CCS-negative groups, no evidence of improvement in test AUC was observed with the inclusion of age and sex.

**Table 3:** Effect of demographic features on model performance

Input features	Test AUC
- Output probabilities of the best-performing deep learning models for each architecture - *No demographic features* are additionally considered	0.95 (0.94–0.97)
- Output probabilities of the best-performing deep learning models for each architecture - *Only age* is additionally considered	0.95 (0.94–0.97)
- Output probabilities of the best-performing deep learning models for each architecture - *Only sex* is additionally considered	0.95 (0.94–0.97)
- Output probabilities of the best-performing deep learning models for each architecture - *Both age and sex* are additionally considered	0.95 (0.94–0.97)

Note: 95% confidence intervals are in parentheses. The term “best-performing model” refers to the model exhibiting the largest AUC in the test set. The architectures studied in the present study are ResNet50, VGG16, MobileNetV3, and EfficientNetV2. *AUC*, area under the receiver operating characteristic curve

## Discussion

Despite the clinical significance of DCM and the increasing popularity of DL in radiology, large cohort studies and extensive comparisons between DL architectures on the task of predicting CCS have not been thoroughly explored in the literature. In our study, we established a large DCM cohort comprising 8676 patients to train a variety of DL models, including ResNet50, VGG16, MobileNetV3, and EfficientNetV2. The ensemble model resulted in the best AUC of 0.96. Grad-CAM analyses have shown that the trained models consistently show sensitive feature-detecting capabilities and agreement with each other.

The patient cohort used in our study is considerably larger than those used in preliminary research on developing DL models for predicting DCM, as most cohorts hardly include more than 1000 patients [10–13]. As this number is not suitable for training deep learning models, which require at least tens of thousands of data points, most preliminary studies were forced to oversample inputs from the same patient, making the dataset highly redundant. However, in this study, we were able to exploit the abundance of data and achieved state-of-the-art AUC and generalizability, comparably improved from previous studies, in which one reported an AUC of 0.94 with axial T2 MRI [12].

Furthermore, unlike previous studies, we experimented with a variety of DL architectures to comprehensively understand the behaviors of different models. In general, they exhibited congruency and a similar level of performance, as represented in Fig. 4a and Fig. 4b, showing representative CCS-negative and positive cases. However, explainability differed in some samples, highlighting the need for model diversity. Figure 5a exhibits multiple CCS at C3-4 and C6-7, and CAMs for ResNet50 and VGG16 are vaguely distributed, while those of MobileNetV3 and EfficientNetV2 are separately focused on the affected levels. This results in a bimodal heatmap of the ensemble model, thereby illustrating the complementary nature and importance of ensembling models.

Figure 5b exhibits diffuse CCS caused by OPLL affecting C2-4. All of the models successfully localize the level of cord compression, but CAMs slightly differ. VGG16, MobileNetV3, and the ensemble model produce heatmaps centered at the thecal sac, but interestingly, EfficientNetV2 precisely pinpoints the causative lesion. Thus, training a single DL architecture may not be enough to specify all abnormal findings present in the input, and it is recommended to take a comprehensive approach that considers the outputs of each model rather than relying solely on a single model.

Figure 5c demonstrates a patient with T2 hyperintensity in C3-6 without definite evidence of cord compression. The output probabilities of the models ranged from 0.20 to 0.59, indicating controversy among models. Grad-CAM analyses imply that the signal abnormality of the spinal cord may have raised the outputs of some models. Therefore, DL models are capable of capturing possibly problematic features from the input images.

Figure 6a demonstrates a sample that produced false negative outputs. The models generally do not have difficulty locating a region with the greatest likelihood of CCS. However, confusion may have been caused by the subtle degree of spinal cord indentation and sufficient CSF space present along the spinal canal. Figure 6b is a test sample that resulted in false positive predictions. In Fig. 6b, models may have generated large output based on the findings at C3-5, where severely progressed cervical spondylosis is identified. Thus, it can be inferred that falsely classified samples are mostly difficult cases that may be controversial even for experts to determine solely from a few mid-sagittal slices. However, considering Grad-CAM analyses, DL models possess high explainability and can potentially guide clinicians.

Despite the strengths of our DL model, there exist some limitations that should be addressed in future work. First, our cohort contains selection bias since the data was retrospectively collected from patients who have undergone an MRI of the cervical spine at a tertiary hospital. Therefore, the prevalence of CCS was 59.0%, much greater than that of the general population, which is less than 0.1% [1, 2]. This may have resulted in the models being overly sensitive to positive findings, raising false positive rate, but it should be considered beneficial in the clinical context because it may contribute to reducing false negative errors. To minimize the possibility of data leakage and further improve generalizability, training and validating models on a large-scale multi-center cohort is necessary. An external set established at separate healthcare institutions may be used to promote a more generalizable evaluation of the model, and this is left for future work.

Another drawback of our study is the inevitable loss of information caused by selecting only three sagittal images for input from each patient. This approach was made to facilitate transfer learning of pretrained models, which generally take three-channel inputs. However, it may have reduced the sensitivity in identifying CCS-positive cases in which the cause does not originate from lateral structures not commonly observed from the mid-sagittal plane, for example, paracentral bulging of intervertebral disc and asymmetric deformity of the spinal canal. This may have resulted in some false negative predictions. Novel architectures that utilize multimodal or multi-channel inputs by incorporating three-dimensional convolutional layers should be thoroughly explored for future work [21–23].

In conclusion, we established a large DCM cohort and successfully developed DL models that predict CCS from mid-sagittal T2-weighted MRI sections. Through extensive analyses, we confirmed that ensembled models result in the best overall performance, but careful examination of the outputs of each model is suggested to effectively assess explainability.

## Data Availability

The dataset generated and analyzed during the current study is not publicly available due to patient privacy concerns and ethical restrictions. However, de-identified data may be made available from the corresponding author upon reasonable request. The source codes for loading and running models are provided in the following GitHub repository: https://github.com/rhee1998/snuh_c_spine_mr_t2sag_ccs.
